# Non-Intrinsic, Systemic Mechanisms of Cellular Senescence

**DOI:** 10.3390/cells12242769

**Published:** 2023-12-05

**Authors:** Rachael E. Schwartz, Irina M. Conboy

**Affiliations:** Department of Bioengineering, University of California Berkeley, Berkeley, CA 94720, USA; reschwartz@berkeley.edu

**Keywords:** aging, cellular senescence, bystander senescence, systemic milieu, blood serum and plasma

## Abstract

Cellular senescence is believed to contribute to aging and disease through the activity of secreted factors that promote inflammation, remodel the extracellular matrix, and adversely modify the behavior of non-senescent cells. While the markers and properties of senescent cells are still under investigation, it is postulated that cellular senescence manifests in vivo as the consequence of cellular damage that accumulates and becomes exacerbated with time. Yet, the notions that senescence has a solely intrinsic and time-dependent nature are questioned by the rapid induction of senescence in young mice and young cells in vitro by exposure to blood from aged animals. Here, we review some of the research on the systemically present factors that increase with age and may contribute to extrinsically induced senescence or “bystander senescence”. These include proteins, reactive oxygen species, lipids, and nucleic acids, which may be present in individual soluble form, in vesicles, and in non-membranous multi-component macromolecules.

## 1. Introduction

In experimental cell culture systems, cellular senescence can be induced by oncogene activation, telomere shortening (replicative exhaustion), or DNA damage (such as that caused by gamma or UV radiation, or chemicals such as doxorubicin) [1,2]. Cellular senescence is considered a cancer avoidance mechanism, as it arrests the cell cycle to terminate replication [3]. Senescence markers thus include the upregulation of cyclin-dependent kinase inhibitors 1A (p21^Cip1/Waf1^) and 2A (p16^INK4A^) [3,4] as well as the phenotypic marker senescence-associated β-galactosidase (SA β-gal) [4,5]. It is postulated that analogous mechanisms result in cell and tissue senescence in vivo and that cell-intrinsic mechanisms of senescence manifest their effects throughout the lifetime, such that senescent cells accumulate with age [6]. However, in mice, senescence can be induced in a very short period of time (two weeks) in a young animal that has had 50% of its blood exchanged for blood from an old animal, which is an effect that can be attenuated by pretreatment of the old animal with senolytics [7]. Moreover, young cells that are cultured with serum from aged animals become senescent in days based on increased p16^INK4A^, p21^Cip1/Waf1^, and SAβ-Gal and diminished laminB1 and proliferation, and old serum dominates over young in vivo [8] and in vitro [7]. The identification of factors produced by senescent cells and carried in the aged systemic milieu that can induce senescence in healthy cells, a process called “bystander senescence” [9], and the mechanisms by which they operate may lead to targeted therapies to alleviate senescence and its adverse consequences. Bystander senescence has been shown by several studies to be induced by the transplantation of senescent cells [10,11]. In this review, however, we focus on those systemic factors that are physiologically elevated in animals upon aging and that induce bystander senescence in healthy cells and tissues. The identification and characterization of these circulatory factors that are elevated with age may entail the discovery of druggable biomedically relevant targets.

The most logical candidates for inducing bystander senescence are the elements of the senescence-associated secretory phenotype (SASP), the term applied to the secretome of senescent cells [12], which have been classified into two broad categories: soluble individual components and those that are associated with membranous vesicles [13]. Certain components of both types have been found to transmit senescence to other cells in culture [14,15,16,17,18,19,20,21,22,23,24], although this has mostly been demonstrated to occur in a juxtacrine or paracrine manner rather than over longer distances in an endocrine fashion. Not yet formally included in the SASP, but also potentially capable of transmitting senescence, are multi-component macromolecules.

In this review, we focus on SASP elements and other factors secreted by senescent cells, both soluble and vesicular, as potential triggers of senescence in healthy cells. We also discuss some candidate macromolecules that are not enveloped by a membrane: vaults, microRNA-Argonaute 2 aggregates, and virtosomes. Finally, we emphasize remaining questions and avenues for future research in the field of transmissible senescence.

## 2. Single Component Soluble Secreted Factors

**Figure 1:**
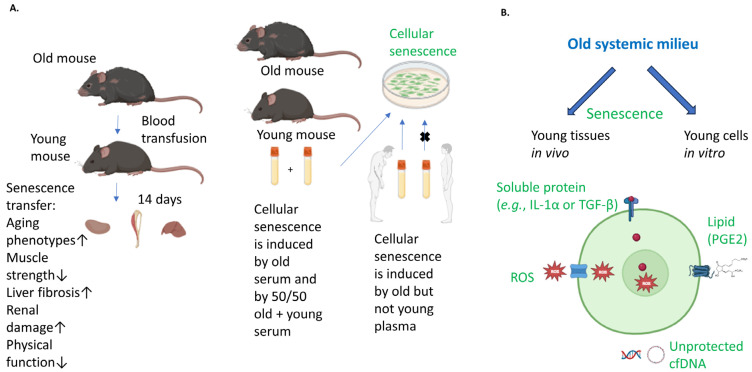
**Schematic on the rapid induction of senescence in young tissues in vivo and young cells in culture by old blood factors and illustration of possible soluble factor mechanisms.** (**A**) A summary of the phenomenon of rapid extrinsic induction of senescence in young tissues and cells. Blood from old mice is transfused to young mice, leading to senescence transfer to kidneys, skeletal muscle, and liver. Serum from old mice, a 50/50 mix of serum from old and young mice, or plasma from old, but not young, humans induce senescence in cell culture. (**B**) Potential endocrine mechanisms. A soluble protein (e.g., IL-1α or TGF-β) or lipid (PGE2) may trigger intracellular signaling upon binding and may also undergo receptor-mediated endocytosis with the possible transport of some proteins to the nucleus. Some ROS can diffuse across the plasma membrane (singlet oxygen, hydrogen peroxide) or use anion channels (superoxide anion) or aquaporins (hydrogen peroxide) to cross the plasma membrane; once inside a cell, ROS may cause damage or serve as a signaling molecule. A method by which sufficient unprotected genomic or mitochondrial cfDNA could be internalized to induce senescence has not yet been shown.

The comprehensive screening of known SASP components and other age-associated factors secreted by senescent cells that may yet be identified (for example, by the NIH Common Fund’s Cellular Senescence Network Program, https://commonfund.nih.gov/senescence (accessed on 30 November 2023)), which are often cell-specific, for their ability to systematically propagate senescence, remains to be performed. A few candidates that have been found to be secreted by some cell types are described below and illustrated in Figure 1 [25,26,27].

Most soluble SASP factors are proteins, and some act through direct cell–cell contacts. Interleukin 1-alpha (IL-1α), for example, can be displayed on the surface of a senescent cell and bind its receptor on adjacent cells, triggering the transcription of target genes of nuclear factor kappa-light-chain-enhancer of activated B cells (NF-κB) and production of the SASP component IL-6 in a juxtacrine manner; many SASP components (including IL-1α) are targets of NF-κB transcription [14]. IL-1α is typically secreted [28,29] and therefore can induce the SASP in non-adjacent cells in a paracrine manner. Paracrine senescence can also be mediated by TGF-β family ligands [30,31], VEGF, CCL2, CCL20 [31] CXCL10/IP-10 [32], and insulin-like growth factor binding protein 3 [33]. IL-1β, which, like IL-1α, binds the IL-1 receptor and activates NF-κB, may also induce bystander senescence [15]. The SASP component tumor necrosis factor alpha (TNF-α) has been shown to induce senescence [34]. TNF-α, in combination with interferon gamma (IFN-γ), has also been used to induce senescence in cancer cells [35]. The transmission of senescence through blood heterochronicity in mice [7] and in cultured cells might be relevant for understanding systemic senescence in people, such as when TNF-α and other SASP proteins become elevated with age [36].

Not all SASP factors are proteins; for example, a lipid component of the SASP, prostaglandin E2 (PGE2) [12], has been found to induce bystander senescence [16]. PGE2 is a mediator of inflammation that is generated by the cyclooxygenase 2 (COX2) conversion of arachidonic acid. Blocking two of the E series of prostaglandin receptors (EP2 and EP4) reduced the incidence of senescence in cells treated with PGE2 [16].

Reactive oxygen species (ROS) produced by senescent cells, primarily in poorly functioning mitochondria, can leak out [12] and induce senescence in nearby cells, in part by the activation of NF-κB [17]. ROS can cause double-strand DNA breaks [37], activating the DNA damage response and NF-κB [38], which if unresolved may lead to senescence.

Cell-free DNA (cfDNA), which can be protected by association with a vesicle or by interactions with other molecules, or unprotected (the latter is referred to in the literature as “naked” cfDNA), has, so far, not been classified as a SASP component. The extent to which naked cfDNA exists in the blood is unclear. Whether naked cfDNA taken up by a cell from the blood would cause bystander senescence is an interesting question. Intrinsic cellular senescence is promoted when DNA enters the cytoplasm from the nucleus in the form of cytoplasmic chromatin fragments (CCFs), as it activates the cyclic GMP–AMP synthase–stimulator of interferon genes (cGAS–STING) pathway [39]. Mitochondrial DNA released into the cytoplasm triggers cGAS–STING signaling [40]. cfDNA originating from mitochondria is known to increase in the blood with age and to be secreted by senescent cells [41]. Nuclear cfDNA also increases with age and can be released from senescent cells often in the form of CCFs [42] or in exosomes [43]. Whether the cfDNA existed as a single-component, unprotected macromolecule, however, is not always clear from the published data; the method of isolation can make it difficult to determine whether the cfDNA was protected when in circulation. Extracellular DNases degrade unshielded DNA [44], which would be anticipated to limit the total amount taken up by other cells absent pathological release into the system. The half-life of cfDNA in the circulation has been estimated at 15 min to 2.5 h [45]. The source of most cfDNA in plasma is hematopoietic cells; however, apoptotic and necrotic cells of other lineages, as well as tumor cells, can also be a source of cfDNA [45]. In the case of necrotic cells, the cfDNA would not be protected by a membrane, although it could be partially shielded by nucleosomes. Thus, while naked cfDNA may, in theory, promote bystander senescence, in practice, it seems unlikely to be present in sufficient quantities to accomplish this result. As discussed below, however, there are forms of cfDNA that are protected from degradation.

## 3. Membrane-Bound Bodies

**Figure 2:**
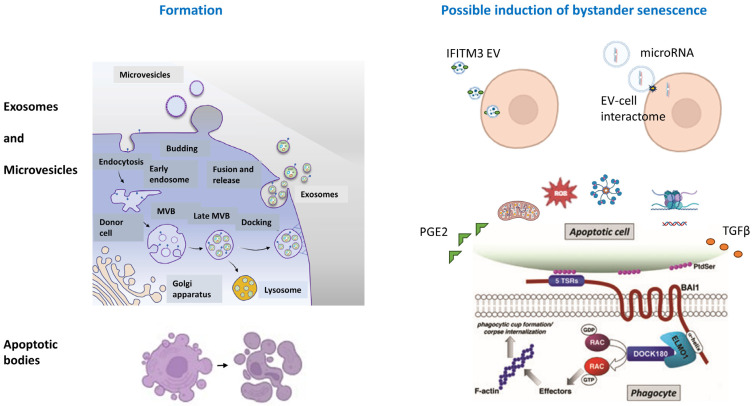
**Exosomes, microvesicles, and apoptotic bodies associated with bystander senescence.** Exosomes are released when multivesicular bodies formed through the endocytic process merge with the plasma membrane and discharge their contents into the extracellular space, while microvesicles bud off the plasma membrane. Cells may take them up by adhesion, membrane fusion, receptor-mediated endocytosis, pinocytosis, or phagocytosis. They may also bind cell surface receptors without being taken up. Exosome or microvesicle components that may induce bystander senescence include the membrane protein IFIT3 (shown), some membrane lipids, and internally carried microRNAs (shown), mRNAs, and proteins. Among the mechanisms of action are signal transduction from receptor binding and effects on transcription and translation. Apoptotic bodies form through a process of disassembly that includes membrane blebbing and fragmentation. NPCs and other cells phagocytose apoptotic bodies employing a process directed by engulfment and cell motility protein 1 (ELMO1), which is involved in cytoskeletal rearrangement; the phagocytosis of apoptotic bodies can result in TGF-β and PGE2 release (shown) and affect transcription and translation. Undigested genomic or mitochondrial DNA in the apoptotic body might also trigger cGAS–STING signal pathways, and other contents (e.g., ROS, apoptotic pathway proteins such as proteases) may have varied effects.

Extracellular vesicles (EVs) have been implicated in the phenomenon of bystander senescence [46], yet understanding how EVs may induce it is more challenging than studying individual soluble candidates, for multiple reasons. One is that vesicles carry a diverse assortment of molecules, any one or more of which may promote or inhibit senescence. EVs are commonly characterized by their size and origins: exosomes, which are the smallest, are created via the endosomal pathway; microvesicles bud off from the cell’s plasma membrane; and apoptotic bodies, which are the largest, are formed by the membrane blebbing of cells engaged in programmed cell death [47]. The number of EVs released by a cell increases with cellular senescence [48]. Bystander senescence may be caused by engaging a cell-surface receptor and signal transduction downstream of EV-receptor engagement. Separately, it may be caused by the intracellular effects of the internal contents of the vesicle. Figure 2 illustrates the main classes of EVs associated with potential mechanisms of inducing bystander senescence [18,49,50,51,52,53,54].

An example of a protein on the surface of an EV that can propagate senescence to a healthy cell is interferon-induced transmembrane protein 3 (IFITM3), which is a component in the membrane of a population of exosomes secreted by certain senescent cells and internalized by other cells. A recent study identified it as a potential mediator of bystander senescence [18]. The authors suggested that the bystander senescence might be related to IFITM3-mediated interferon pathway signaling, but the did not exclude the possibility that other components of the EV, such as DNA, RNA, or lipids, could also play a role [18].

The bystander effects of vesicles produced by senescent cells experiencing high ROS levels remain unclear. In oxidative stress, the balance between production and neutralization of ROS is lost, yielding an excess of oxidants [55], which may cause cellular senescence and changes in EV contents. Some vesicular lipids, including those that are increased with higher cellular ROS, can promote bystander senescence. The very long-chain C24:1 ceramide is elevated with oxidative stress and is significantly higher in vesicles found in serum from older women compared to younger ones [22]. When vesicles from young mice were loaded with C24:1 ceramide and incubated with mouse bone marrow stem cells, SAβ-gal was robustly increased [22]. Oxidized phospholipids (oxPLs) on the surface of vesicles released by senescent cells can bind Toll-like receptor 4 (TLR4) and activate NF-κB signaling in bystander cells [56]. TLR4 is a mediator of multiple cellular responses that become altered with age, including inflammation [57]. Notably, in the age-specific studies on youthful normalization of the systemic proteome after therapeutic plasma exchange (TPE), TLR4 was identified as a nodal point for age-altered and TPE-rejuvenated protein networks [58].

Interestingly, some ROS-stressed EVs of senescent cells contain antioxidants that might protect nearby cells and even attenuate their experimentally induced senescence [59]. However, another study suggested that vesicles of ROS-stressed cells cultured at 21% oxygen that had high SAβ-gal increased the SAβ-gal in cells cultured at a 3% oxygen level. The EVs from cells cultured at 21% oxygen had lower levels of microRNA-302b (miR-302b) than those from cells cultured at 3% oxygen with miR-302b being a potential trigger for delaying senescence [19]. These results point to the need for more work, using multiple assays to measure senescence, to determine the effect of oxygen levels on EV-promoted and/or reduced bystander senescence.

Support for the notion that microRNAs (miRNAs) in vesicles from oxidatively stressed cells may promote ROS production and bystander senescence in non-senescent cells was provided when HUVECs were chemically induced to produce the superoxide anion and their EVs were isolated. The superoxide anion and NF-κB activation were significantly increased in endothelial progenitor cells (EPCs) treated with these EVs compared to EPCs treated with vesicles from control HUVECs. Profiling of the miRNA content of the two populations of vesicles revealed a differential expression of 23 miRNAs, including elevated miR-181a-5p and miR-4454 in vesicles from the treated cells. These two miRNAs had been previously found to be linked with NF-κB signaling [21].

Given that miRNAs are involved in the regulation of gene expression, it is perhaps not surprising that vesicles from senescent cells containing miRNAs broadly promote bystander senescence. For example, EV-delivered miR-34a-5p (miR-34a), which targets the cyclin-dependent kinase inhibitor 1A (p21^Cip1/Waf1^), was implicated as a mechanism by which senescent chondrocytes from osteoarthritis patients induce senescence in nearby chondrocytes [60]. (In the context of microRNAs, “5p” refers to the mature miRNA originating from the 5′ arm of the precursor product; the other arm is designated “3p”. The role of the 3p strand of miR-34 is not as well-known [23].) miR-34a has been found to promote senescence in other tissues, such as the skeletal muscle, heart, and brain, potentially targeting the Wnt and Notch pathways, as well as pro-survival factors such as Sirt1 and Bcl-2 [61,62]. The microRNA miR-433 has been shown to induce cellular senescence in ovarian cancer cells [24] and multiple myeloma cells [63] via the downregulation of cyclin-dependent kinase 6, and cells producing the highest level of miR-433 also showed increased SAβ-gal after chemotherapy [24]. The miR-433 in those studies was present in exosomes [24]. Moreover, miR-433, along with other miRNAs, has been found to be upregulated with age in human dental pulp cells [64]. Two miRNAs, miR-21-5p and miR-217, were upregulated in senescent HUVECs and human aortic endothelial cells (HAECs) as well as in small extracellular vesicles secreted by these senescent cells [65]. The locus coding for miR-21-5p showed DNA demethylation in the region surrounding its transcription start site and RNA over-expression consistent with the upregulation of transcription [65]. The treatment of non-senescent cells with small vesicles secreted by the senescent cells also significantly reduced the expression of DNMT1 and SIRT1 and increased the presence of senescence markers [65].

While much of our knowledge of the role of miRNAs in bystander senescence is based on in vitro or mouse studies, analysis of small EVs isolated from the plasma of young, elderly, and centenarian human donors confirms the relevance of miR-21-5p levels by showing that it follows an age-related inverted U-shaped trend [65]. Further studies of human subjects will likely produce additional prospects for inducers of bystander senescence and elucidation of the pathways by which they function.

Both nuclear and mitochondrial DNA can be associated with exosomes and microvesicles from senescent cells [29,43], and apoptotic bodies contain fragmented DNA [66]. Viral DNA can also be contained in vesicles from infected cells [43]. The induction of senescence by vesicular DNA appears to be not well studied to date. However, of the various types of vesicles, apoptotic bodies would carry the largest quantities of DNA, and apoptosis increases with age in some tissues, including the brain [67,68], skeletal muscle [69], and heart [70,71]. Moreover, the load of some virally infected cells and their elimination by cytotoxic immune cells (CD8+ T cells and NK cells) is expected to increase with age [72], with a concomitant increase in circulating apoptotic bodies. Another contribution to this age-associated increase would come from cells that undergo intrinsic apoptosis, rather than senescence, as a consequence of age-associated stress [73]. Furthermore, although senescent cells are resistant to apoptosis, they do not always avoid it entirely [73,74,75,76].

Although professional phagocytes (such as macrophages and immature dendritic cells) generally engulf and degrade apoptotic bodies, other cells can also take them up [77], potentially leading to bystander senescence. Some neuronal precursor cells (NPCs) can uptake apoptotic bodies [50]. Other reported non-professional phagocytic cells include hepatic stellate cells [78], airway epithelial cells [79], and retinal pigment epithelial cells [80]. The ability of a variety of cells to take up apoptotic bodies opens the possibility of the widespread transmission of senescence.

It is also likely that senescence-promoting vesicles and soluble factors are present in the systemic milieu simultaneously and might operate through positive feedback. For example, active phagocytes engulfing apoptotic bodies secrete TGF-β, a mediator of bystander senescence, which is also overproduced by many damaged tissues; at youthful levels, TGF-β attenuates major histocompatibility complex (MHC) expression and immune responses, but at age-elevated levels found in old blood serum, it promotes inflammaging and suppresses tissue regeneration [77,81,82]

## 4. Non-Vesicular Multi-Component Macromolecules

In addition to individual molecules and vesicles, a third form of secreted factors consists of complexes of different molecules that are not surrounded by a membrane. We focus here on macromolecules that are consistently formed from the same types of components. However, we note that aggregates of dysfunctional molecules, such as misfolded proteins, sometimes thought of as cellular garbage, may also play a role in bystander senescence [83].

One such multi-component macromolecule is the nucleoprotein body known as a “vault”: a hollow, barrel-shaped structure whose function is still not well-defined. The largest component of these macromolecules is major vault protein (MVP) [47]. Mammalian vaults consist of MVP, two minor vault proteins, vault poly (ADP-ribose) polymerase, telomerase-associated protein-1, and three small vault RNAs [84]. The production of MVP increases with age, and endogenous MVP contributes to apoptosis resistance in senescent cells by upregulating the anti-apoptotic protein Bcl-2 [84]. The secretion of MVP increases with senescence in normal oral keratinocytes [29]. Vaults can be taken up by cells and have been considered as potential drug delivery vehicles [85]. Accordingly, it would be interesting to know whether vaults taken up from the systemic milieu may contribute to bystander senescence in healthy cells. The upregulation of Bcl-2 and other anti-apoptotic molecules in the same family is commonly observed in cellular senescence [86], so MVP in vaults taken up by cells may also increase the production of these anti-apoptotic proteins.

miRNAs can also be found in aggregates that protect them from degradation by virtue of their association with argonaute 2 (Ago2) or high-density lipoprotein (HDL)-associated proteins [87]. The Ago2 protein combines with a small RNA to compose the core of the RNA-induced silencing complex (RISC) that represses translation or enhances the degradation of target mRNA [88]. (In addition to its function in the RISC, the translocation of endogenous Ago2 to the nucleus facilitates cytokine-induced senescence [35].) There is, however, a dispute in the field concerning the relative percentages of vesicle-enclosed and non-vesicular miRNAs. Some researchers take the position that the majority is non-vesicular [89], and others take the position that the majority is concentrated in exosomes [90].

cfDNA can be packaged into DNA–RNA–lipoprotein particles that have been designated “virtosomes” [91,92]. The proteins include DNA-dependent DNA and RNA polymerases [92]. The uptake of virtosomes by normal cells exposed to blood plasma from cancer patients may be responsible for oncogenic transformation [92,93]. Whether the uptake of an oncogene in this manner could instead trigger oncogene-induced senescence in some cells appears to be not yet addressed.

**Figure 3:**
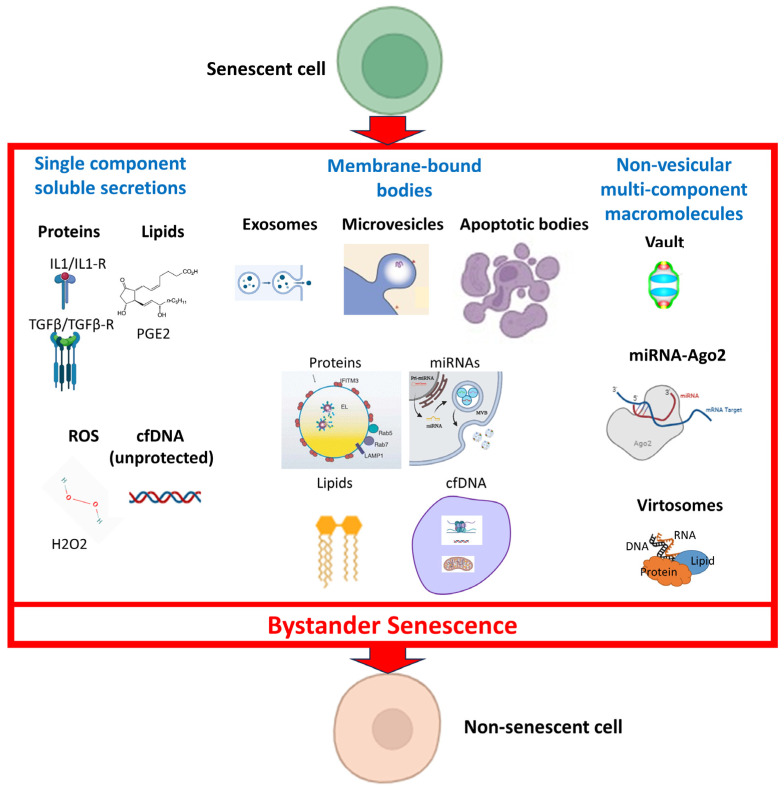
**Summary of the potential mechanisms of propagation of senescence.** The three major categories of possible transmitters of bystander senescence discussed in this review (single-component soluble secretions, membrane-bound bodies, and non-vesicular multi-component macromolecules), their respective subcategories, and examples of each, are shown.

Figure 3 provides an overview of the above-discussed potential propagators of senescence [53,94,95,96,97].

## 5. Conclusions

Current data suggest that senescence is neither entirely intrinsic nor simply time-dependent. Certain soluble elements present in the systemic milieu—proteins, lipids, and ROS—can induce bystander senescence, but there are likely others, as yet unidentified, that are also capable of accomplishing this result, either individually or in combination. EVs can induce bystander senescence, but only a few components of these vesicles that are responsible for this result are specifically known. Many of these components are miRNAs; however, hundreds of miRNAs have been identified, and we still have much to learn about their functions. Whether nuclear or mitochondrial DNA in apoptotic bodies contributes to senescence in the healthy cells that engulf them is a tantalizingly unexplored frontier. The same is true for non-vesicular multi-component macromolecules that are known to be taken up by non-senescent cells. Our knowledge of the systemic components that provoke senescence in healthy cells remains incomplete, and the importance of this paradigm demands further investigation.

The characterization of age-altered contents—proteins, lipids, and nucleic acids—of vesicles and aggregates, as well as the identification of the senescent cells that release them and the mechanisms of their uptake, is an enormous but essential endeavor. A deeper understanding of this field will be invaluable to the development of senolytics that can prevent an organism-wide loss of health due to the propagation of senescence from a pathological tissue to healthier tissues. Notably, it is important to confirm the many in vitro studies using in vivo paradigms. In vivo studies will not only confirm the physiological relevance of senescence but also provide insights into whether senescence is induced directly by interactions between the inducing factor and the target cell or mediated indirectly by other cell types (e.g., macrophages and microglia) whose secretions may change when they are affected by the inducing factor. Moreover, it is necessary to employ multiple assays to reach reliable conclusions. When asserting that a cell is senescent, for example, this should be demonstrated by morphology, a lack of proliferation, an increase in cyclin-dependent kinase inhibitor production, SAβ-gal, phosphorylated histone γH2AX, and a change in secretions, or a comparable set of assays appropriate to the cell type and senescence classification. Armed with such information, we should be able to develop new strategies that will inform us regarding physiological senescence, helping to ameliorate the adverse systemic effects of damaged tissues on an organism.

## Data Availability

Data is contained within the article.
